# Supplementary material for: Updated Virophage Taxonomy and Distinction from Polinton-like Viruses
Source: Biomolecules. 2023 Jan 19;13(2):204. doi: 10.3390/biom13020204 (PMC9952930; doi:10.3390/biom13020204)
Supplement: Supplementary file 1 [file biomolecules-13-00204-s001.zip › Virophage_Supplementary_Figures 1-17_Nov.pdf]

Supplementary Figures

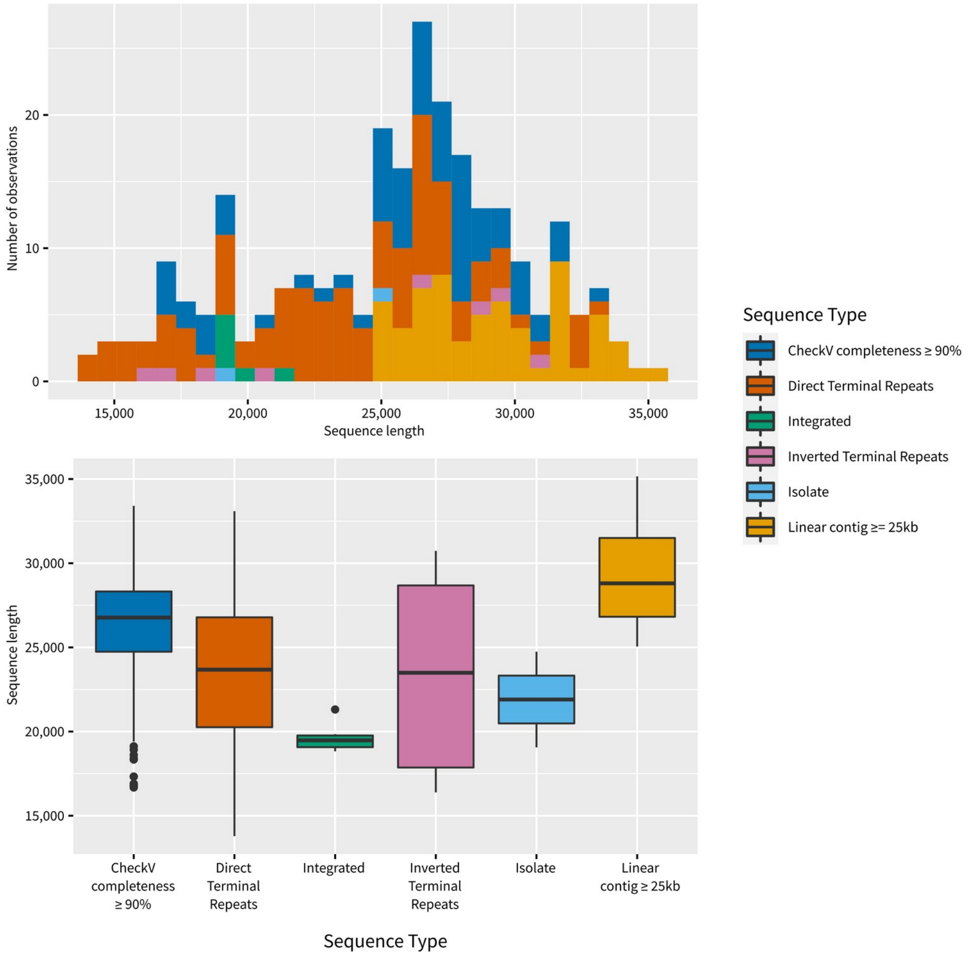

Supplementary Figure S1. Length distribution of complete and near-complete virophage genomes. Sequences were considered as complete or near-complete if they came from isolates, if direct or inverted terminal repeats were detected, if they were integrated in a protist genome with both upstream and downstream host regions, if they were predicted to be ≥ 90% complete by CheckV, or if they were larger than 25kb otherwise.

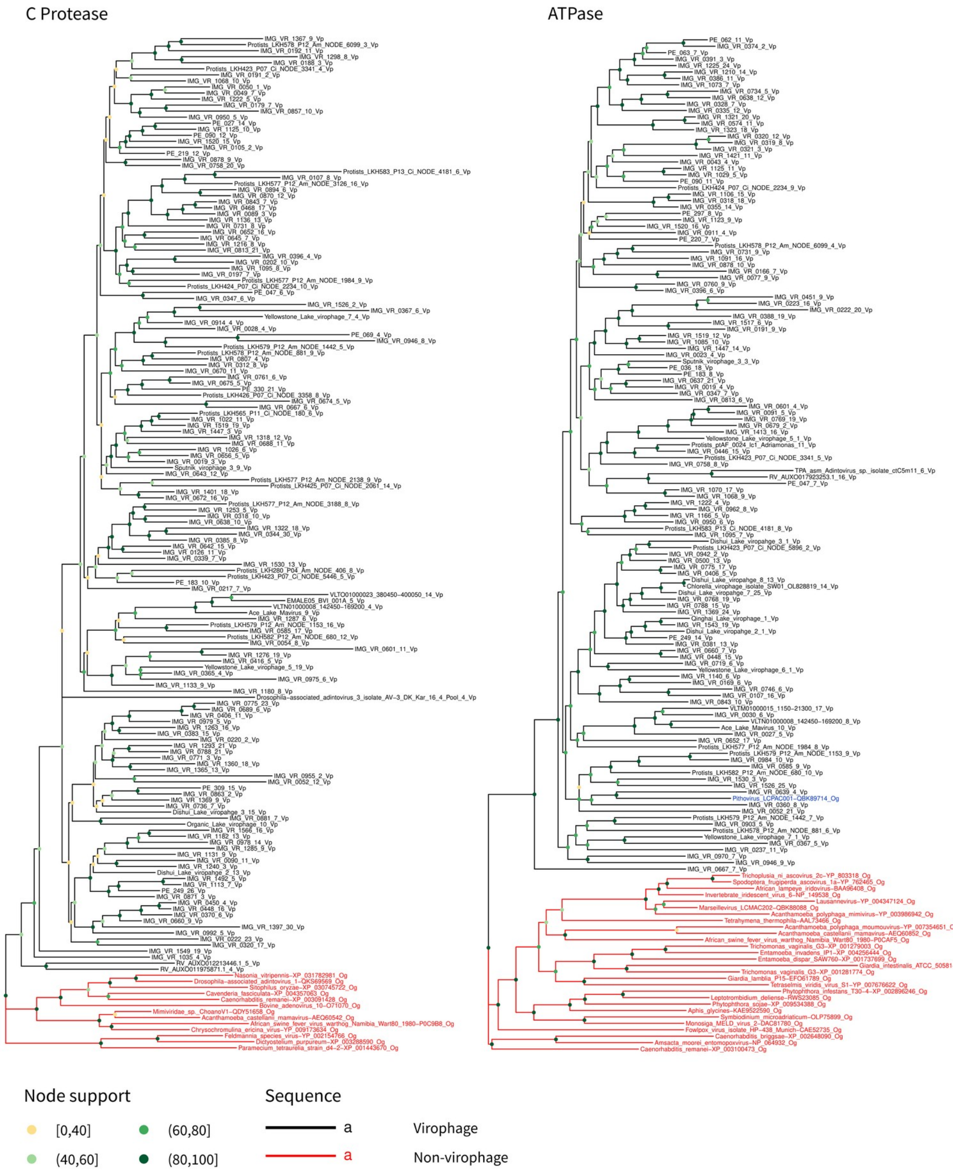

Supplementary Figure S2. ATPase and Cysteine Protease (PRO) trees including virophage and outgroup sequences. For each marker gene, non-virophage sequences were recruited based on outgroups used in a previous study [20] and on *de novo* searches of the NCBI nr database. Input sequences were clustered at 80% amino acid identity prior to alignment and tree building. Outgroup (i.e. non-virophage) sequences are indicated with red branches. The name of the sequence of uncertain origin identified from a publicly available MAG is colored in blue.

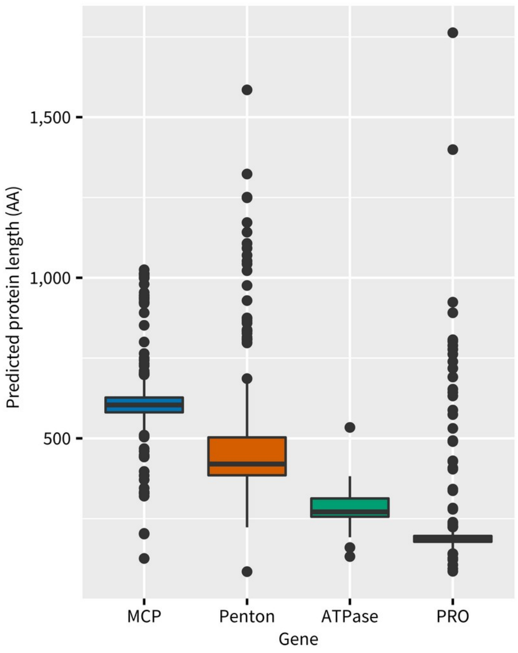

Supplementary Figure S3. Length distribution for the 4 virophage conserved morphogenesis genes. The length of the predicted protein (in amino acid) is indicated for each gene (x-axis). These data only include the predicted proteins from complete and near-complete virophages. Gene names are similar as Fig. 1 A.

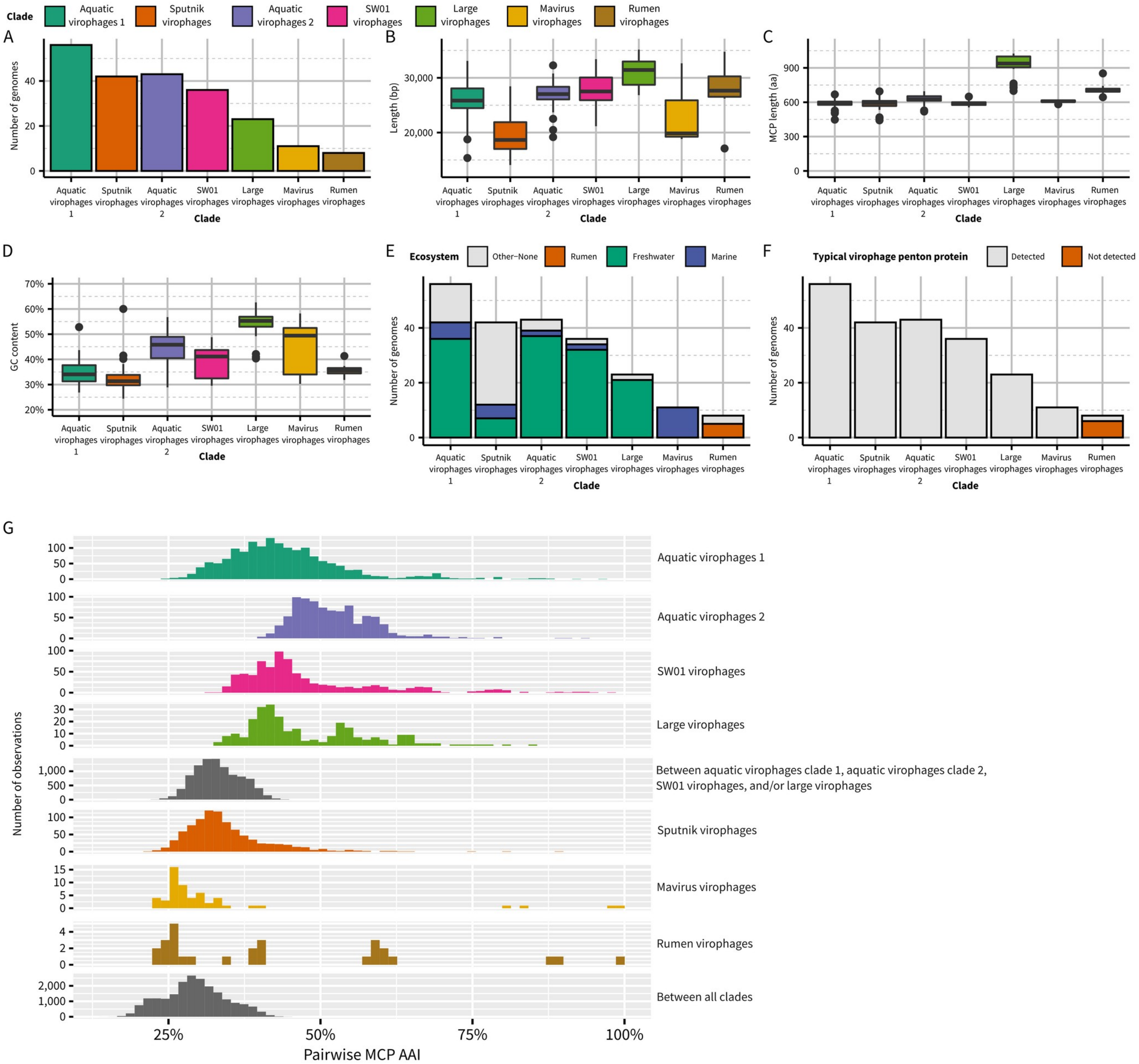

Supplementary Figure S4. Characteristics of proposed virophage clades. Bar charts and boxplots are colored by clade except for the ecosystem panel (E) and penton detection (F), for which bar charts are colored by ecosystem of origin or by detection of the penton protein, respectively. The distribution of pairwise AAI for the MCP sequences within and between core members of the clades is displayed in panel G.

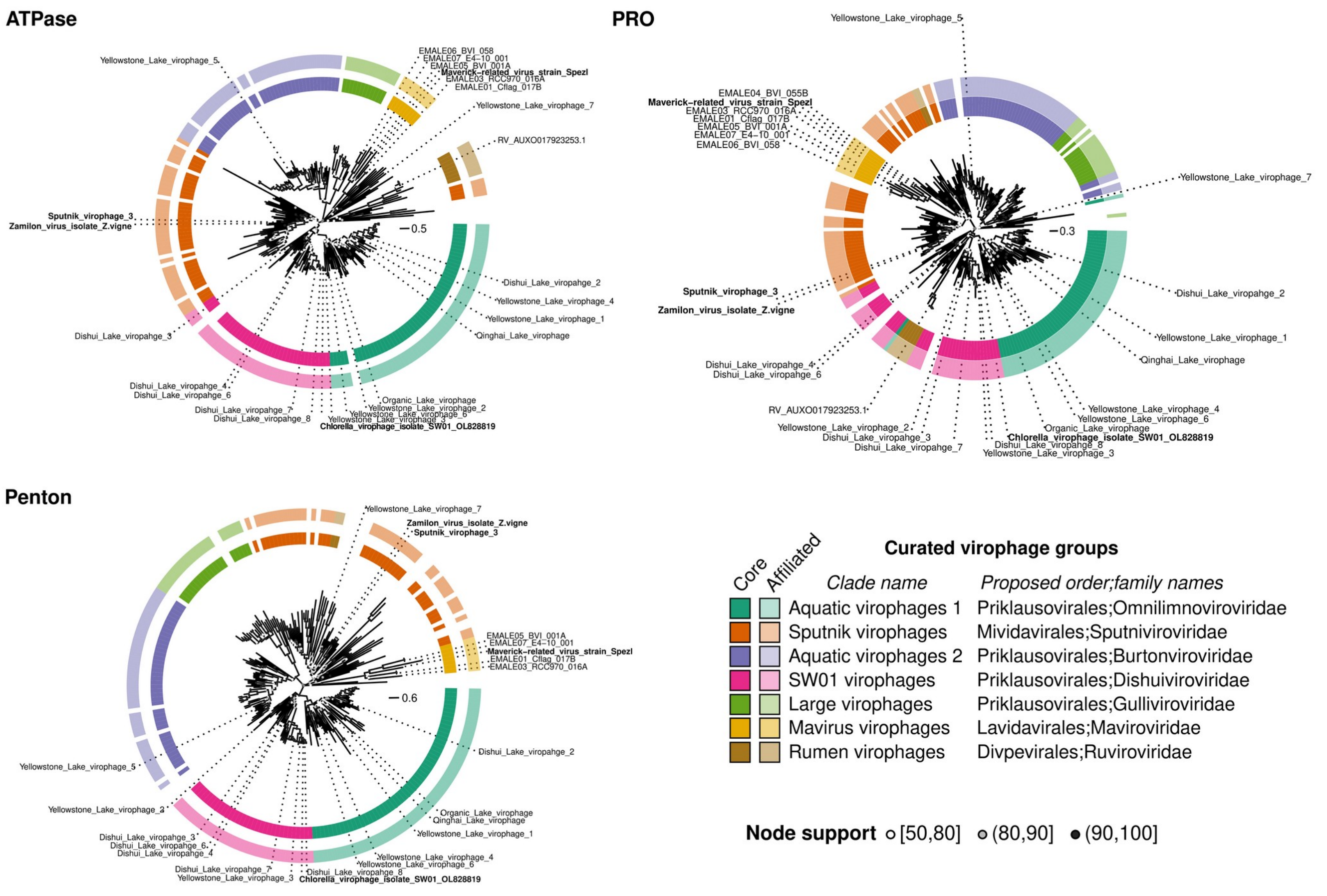

Supplementary Figure S5. Distribution of curated virophage groups on phylogenies built from other virophage markers. For each tree, the corresponding marker gene is indicated on top left, and the classification of each genome is indicated as in Fig. 3. Only complete and near-complete genomes were used to build the trees.

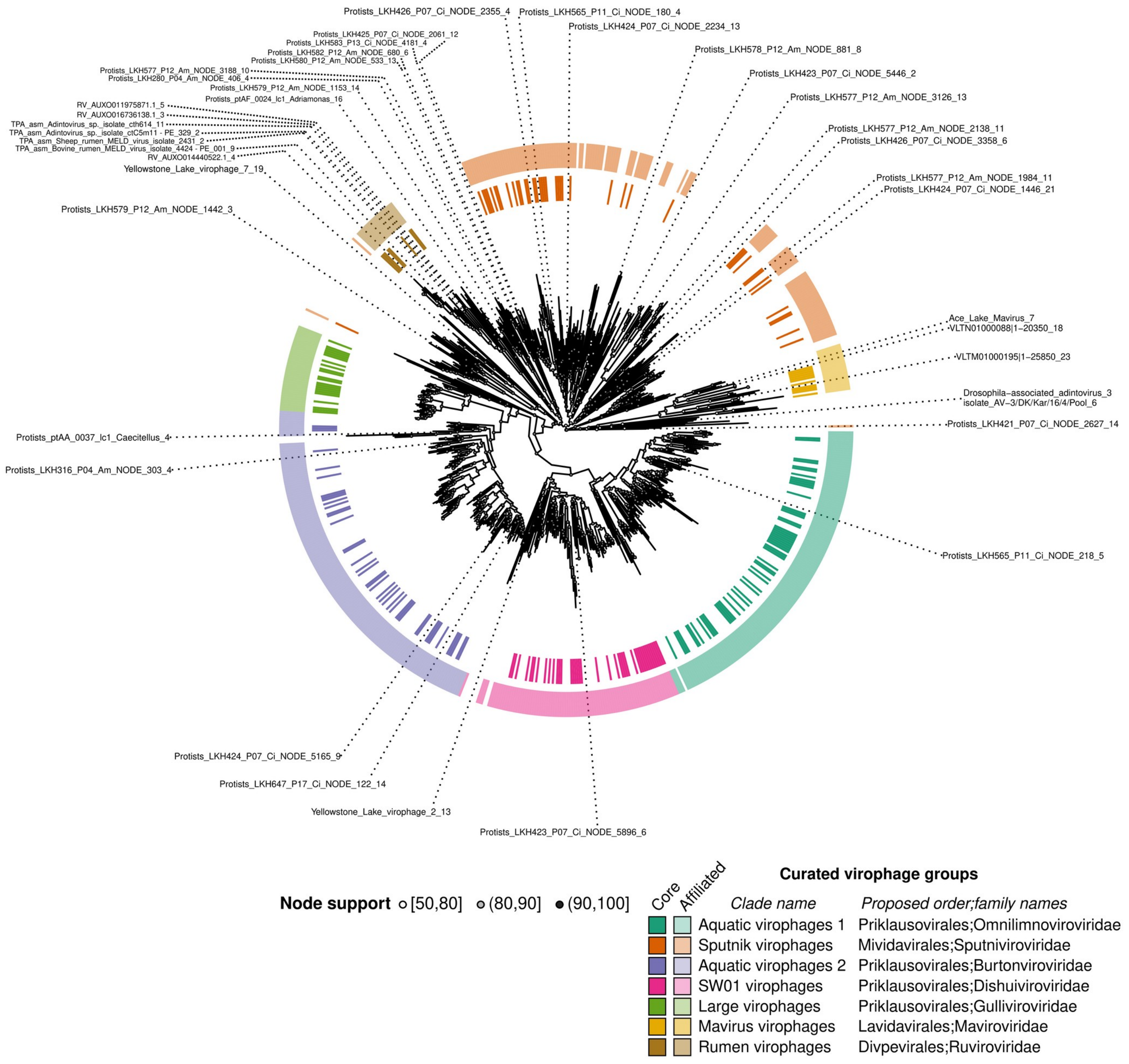

Supplementary Figure S6. Virophage phylogeny (MCP) including complete, near-complete, and partial genomes. As in Fig. 3B, the classification of each contig is indicated via the outer rings, first in dark color if the contig was part of one of the "core" clade, then in light color if the contig was affiliated based on a BlastP hit to a reference (see Fig. 4A and Methods). The name of protist contigs, adintoviruses, and other NCBI-derived references that were not part of a core clade are indicated on the tree.

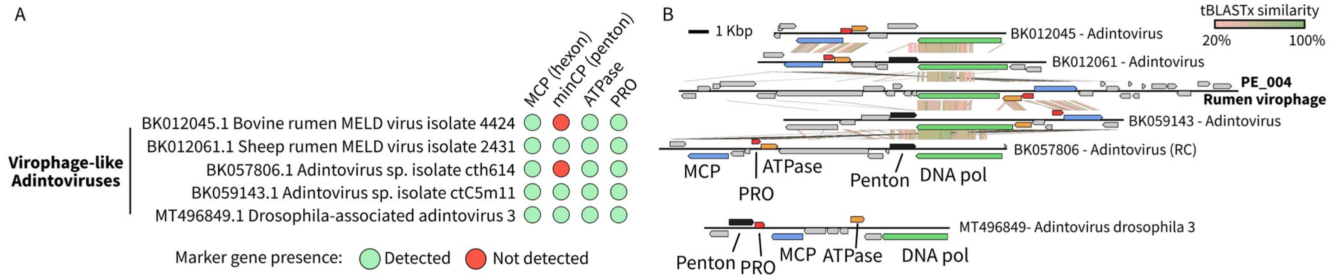

Supplementary Figure S7. Gene content and genome maps of 5 Adintoviruses classified in the *Maveriviricetes* class and closest reference. Panel A displays the detection of virophage marker genes, as in Fig. 1. MCP (hexon): Virophage major capsid protein. minCP (penton): Virophage "minor" capsid protein. ATPase: FtsK-HerA family DNA-packaging ATPase. PRO: maturation Cysteine Protease, Adenain. Panel B includes genome map comparisons for the 4 adintoviruses similar to rumen virophages (top) and the adintovirus similar to Sputnik virophages (bottom). For BK057806, a penton protein was not detected using the HMM profiles built here, but a putative penton could be identified based on similarity to other adintoviruses (panel B).
